# Healthy efficacy of *Nostoc commune* Vaucher

**DOI:** 10.18632/oncotarget.23620

**Published:** 2017-12-22

**Authors:** Zhuoyu Li, Min Guo

**Affiliations:** ^1^ College of Life Science, Shanxi University, Taiyuan 030006, China; ^2^ Institute of Biotechnology, The Key Laboratory of Chemical Biology and Molecular Engineering of Ministry of Education, Shanxi University, Taiyuan 030006, China; ^3^ Institute of Biomedical Sciences, Shanxi University, Taiyuan 030006, China

**Keywords:** Nostoc commune Vaucher, composition, functional food, human health

## Abstract

*Nostoc commune* Vaucher, a macroscopic cyanobacterium, has long been appreciated as a healthy food and traditional medicine worldwide. Accumulated evidence has demonstrated that it possesses a wide range of remarkably protective physiological and pharmacological activities, largely based on animal and *in vitro* studies. In this review, we summarise and update evidence regarding the chemical composition and nutritional characteristics of *Nostoc commune* Vaucher, and comprehensively discuss the recent studies on the antioxidative, anti-inflammatory, anti-carcinogenic and immune regulation properties of *Nostoc commune* Vaucher and *Nostoc commune* Vaucher-derived extracts. The available results demonstrate the potential of it to act as a functional food for the amelioration of human associated diseases. More details from human clinical trials should be a matter of further investigation.

## INTRODUCTION

**Figure 1:**
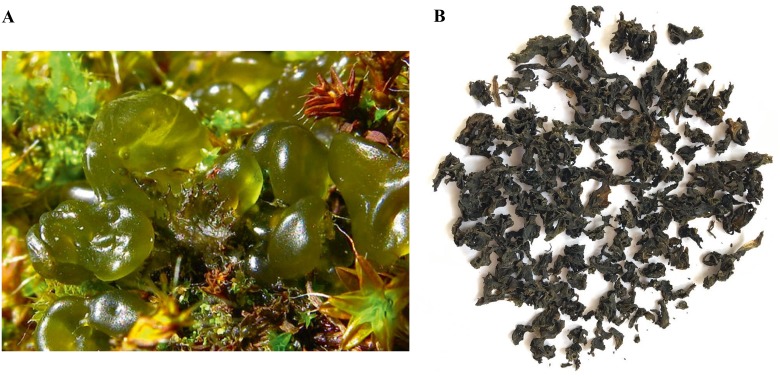
*Nostoc commune* Vaucher in nature (**A**) [1] and under dry condition (**B**).

*Nostoc commune* Vaucher (*N. commune* for short), also known as *Nostoc commune*, is a macroscopic blue-green algal species, which is a member of the genus *Nostoc* (Figure 1A) [1]. Notably, it is easily confused with two other species: *Nostoc flagelliforme* and *Nostoc sphaeroides* Kütz, especially in primary exploration and determination [2–4]. It often forms visually extended mucilaginous layer colonies on soil and is commonly found on stones and mud in freshwater systems [4–6]. When wet, *N. commune* is bluish green, olive green, or brown in appearance; however, under dry conditions, it becomes an inconspicuous brownish mat (Figure 1B) [2]. Amazingly, colonies of *N. commune*, which are naturally subjected to regular cycles of desiccation and hydration, can retain viability for more than 100 years upon desiccation [7, 8]. *N. commune* has been found to be distributed in tropical soil, temperate zones, and both northern and southern polar zones [9, 10]. It has been reported and consumed in many countries and regions especially in Asia. People in some districts of China have widely and traditionally utilized this alga as a food delicacy or ingredient in Chinese medicine since the Eastern Jin Dynasty (317–420 AD), as recorded in the Compendium of Materia Medica (Bencao Gangmu) [2, 6, 10, 11].

**Figure 2:**
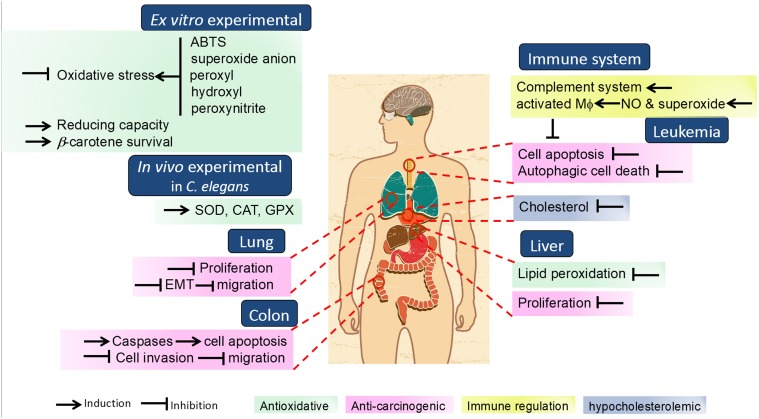
Schematic representation of *N. commune* and *N. commune*-derived extracts regulative properties on human health

Cyanobacteria have been proven to be an extremely valuable source of natural drugs and agents for developing not only medicines, but also functional foods for disease prevention and health promotion [12, 13]. Considerable attention has therefore been devoted to the development and utilization of more effective and non-toxic novel alternatives of natural origin. Currently, there is a sizable body of literature demonstrating *N. commune* and *N. commune*-derived extracts’ ability to develop anti-inflammatory effects, including anti-infectious and antibacterial activity (Table 1), antioxidative prosperities (Table 2), anti-cancer activity, immunomodulation (Table 3) and serum cholesterol reduction. The difference, even diversity, obtained from different studies could be due to the orientation of *N. commune* and experimental methods, as well as the determination and analysis of the measurement results. Unfortunately, to date, the colonies of *N. commune* available in present studies are mainly field materials harvested from natural localities in contrast to limited information on the artificial culturing [5]. However, with the recent development of technology and equipment for culturing, it has become possible to obtain a sufficient sample to conduct more basic research and meet the requirements of industrial-scale applications. In this review, available knowledge regarding the chemical composition and nutritional characteristics of *N. commune* and the healthy efficacy in human associated diseases is summarised and discussed (Figure 2).

**Table 1:** Anti-inflammatory and antibacterial properties of *N. commune*

	Bioactives	Key effects	Ref.
petroleum ether, acetylacetic ester, acetone, ethanol, carbinol & aqueous extracts		1. Lower polar ethyl acetate and acetone extracts exhibited potent antibacterial activity against *P. aeruginosa*, compared to these of *Eschenchia coil*, *Ahernaria alternate*, *Peniicillium sp* and *Trichothecium roseum*. 2. Higher polar methanol and ethanol extracts exhibited potent antibacterial activity against *Alternaria altemata*, *Peniicillium sp* and *Trichethecium roseum*, compared to these of *Eschenchia coil*, *P. aeruginosa* and *Ahernaria alternate*.	[28]
methanol extracts		1. Obvious inhibition growth of *Escherichia coli*, *Staphylococcus aureus*,*Bacillus subtilis*, *Monilia albican* and *Serratia marcescens* (MIC: 1.5–1.0 mg/mL).	[29]
		1. Significant antibacterial activity against *Bacillus subtilis*. 2. No effect against *Salmonella arizonae* and *Proteus mirabilis*.	[30]
fat-soluble compositions		1. Inhibitory effects against six strains: *Escherichia coli* (MIC: 64 μg/mL) >*Staphylococcus aureus > Bacillus subtilis > Aspergillus nigere > Saccharomyces cerevisiae > Pseudomonas stutzeri*.	[31]
diterpenoid	Noscomin	1. Antibacterial activity against *Staphylococcus epidermidis* (MIC 8 ppm),*Bacillus cereus* (MIC 32 ppm), and *Escherichia coli* (MIC 128 ppm).	[23]
	Comnostins A-E	1. Comnostin C had a MIC value for *Escherichia coli* equal to tetracycline, and comnostin E for *Staphylococcus epidermidis* equal to chloramphenicol. 2. Moderate antibacterial activity of comnostins A-E against *Bacillus cereus*, comnostins A-D against *S. epidermidis*, and comnostins A, B, and D against *E. coli*.	[24]
diterpenoid		1. Selective potent antibacterial activity against *Staphylococcus epidermidis* equal to chloramphenicol (MIC 4 ppm). 2. Moderate antibacterial activity against *Bacillus cereus* (MIC 128 ppm).	[25]
anthraquinone		1. Moderate antibacterial activity against *Staphylococcus epidermidis* and *Bacillus cereus* (MIC 32 ppm).	[25]
indane derivative		1. Moderate antibacterial activity against *Staphylococcus epidermidis* (MIC 128 ppm) and *Bacillus cereus* (MIC 64 ppm).	[25]
polysaccharides		1. Dose dependent inhibited growth of *Escherichia coli*, *Anthrax bacillus* and *Staphylococcus aureus*.	[65]
		1. Inhibition of the growth of *Escherichia coli* and *Bacillus subtilis* (MIC:150–250.μg/mL).	[46]
		1. Potent antibacterial activity against *Escherichia coli* (MIC: 50 μg/mL),*Bacillus subtilis* (MIC: 50 μg/mL) and *Serratia marcescens* (MIC: 25 mg/mL). 2. Significant antifungal activity against *Aspergillus nigere* and *Candida albicans*(MIC: 100 mg/mL).	[32]
total flavonoids		1. Dose dependent antibacterial activity against *Escherichia coli* and *Staphylococcus aureus*. 2. Dose dependent antifungal activity against *Aspergillus nigere* and *Candida albicans.*	[34]
lipopeptide	Nostofungicidine	1. Potent antifungal activity against *Aspergillus candidus* (MIC: 1.6 μg/mL).	[33]
heteroglycan	Nc-5-s	1. Less secretion of IL-6 and more of IL-10 in LPS-stimulated human THP-1 monocytes. 2. Anti-inflammatory response through the ERK1/2 pathway and/or the Akt/PI3K pathway.	[13]
indole alkaloid	Reduced-scytonemin	1. Suppression LPS/IFNγ-induced NO production in murine macrophage RAW264 cells by inducing HO-1 expression via the Nrf2/ARE pathway.	[8]
b-ionone derivatives	Nostocionone and its derivatives, NostD3	1. Significant inhibition of *Propionibacterium acnes* growth. 2. Suppression heat-killed NO production through decreased iNOS in murine macrophage RAW264 cells (RCB 0535), following inactivation of NF-κB.	[35]

Abbreviations: PI3K, phosphoinositide 3-kinase; NO, nitric oxide; HO, hemeoxygenase; iNOS, inducible nitric oxide synthase.

**Table 2:** Antioxidative properties of *N. commune*

	Bioactives	Key effects	Ref.
polysaccharides		1. O_2_^·−^ scavenging capacity value was higher by 54.8% and 103.7%, respectively, compared to *Nostoc flagelliforme* and *Nostoc sphaeroide*s Kütz.	[43]
		1. Increase the antioxidase activity in *Caenorhabditis elegans*. 2. Decrease the lipid peroxidation level in *Caenorhabditis elegans*. 3. Paraquat-induced oxidative damage reducation in *Caenorhabditis elegans*.	[6]
total flavonoids		1. Higher antioxidative ability than those of polysaccharides and fat-soluble components.	[49, 51]
glycosylated mycosporine-like amino acids (MAA)	1050-Da MAA	1. Approximate 27% contribution of the total radical scavenging activity in the water extract of *N. commune.*	[17]

**Table 3:** Anti-carcinogenic and Immunomodulating property of *N. commune*

Bioactives	Model	Key effects	Ref.
Anti-carcinogenic property			
crude extracts	A549, human lung epithelium adenocarcinoma SMMC-7721, human hepatocellular carcinoma	1. Significant inhibition of cell proliferation with IC_50_ of 24.79 and 51.33 μg/mL, respectively.	[55]
reduced-scytonemin	Human T-lymphoid cell line Jurkat cells	1. Induction of autophagy by ROS formation	[7]
synthesized Nostocionone derivative	Human T-lymphoid cell line Jurkat cells	1. Potently inhibited cell growth more than Nostocionone. 2. Inhibited cell apoptosis through mitochondria via the release of Endo-G.	[4]
water stress proteins	DLD1, HCT-116, HT-29, and SW480, human colon carcinoma	1. Inhibited cell proliferation with IC_50_ of 0.19 ± 0.02, 0.21 ± 0.03, 0.39 ± 0.05, and 0.41 ± 0.01μg/mL, respectively. 2. No effect on normal human intestinal epithelial FHC cells. 3. Halted the cell cycle by G_1_/S arrest. 4. Induced cell apoptosis through caspase-dependent pathway.	[57]
	BALB/c mice with subcutaneously implanted DLD1 cells	1. Suppressed tumor after WSP1 treatment	[57]
	DLD1, human colon carcinoma	1. Increased cell-cell adhesion and reduced cell-matrix adhesion.	[58]
recombinant water stress proteins 1	SW480, human colon carcinoma	1. Significant suppression of cell growth. 2. No effect on normal human intestinal epithelial FHC cells.	[56]
extracelluar polysaccharides	MCF-7, human breast cancer; DLD1, human colon carcinoma	1. Significant inhibited cell proliferation with IC_50_ of 67 and 110 μg/mL, respectively. 2. Induced cell apoptosis through intrinsic, extrinsic and ERS-mediated signaling pathways.	[61]
	NCI-H446 & NCI-H1688, human small cell lung cancer	1. Remarkable suppressed cell migration through blocking the EMT. 2. Inhibition of integrin β1/FAK signaling through regulating cell-matrix adhesion. 3. Blockage of STAT3 nuclear translocation and JAK1 signaling.	[62]
Immunomodulating property			
oligosaccharides	Sheep erythrocytes	1. Strong effect on the complement system by complement fixation test.	[11]
polysaccharides-rich extract	Human peripheral blood-mononuclear cells; U937, human leukemia; Raw 264.7, murine macrophage cells	1. Effective inhibited cell growth of U937. 2. Triggered monocytic/ macrophagic differentiation in U937. 3. Upregulation of GM-CSF and IL-1β and downregulation of IL-6 and IL-17. 4. Increased NO and superoxide secretion in Raw 264.7.	[64]

Abbreviations: ROS, reactive oxygen species; Endo-G, endonuclease G; ERS, endoplasmic reticulum stress; EMT, epithelial-mesenchymal transition program; GM-CSF, granulocyte/macrophage-colony stimulatory factor; NO, nitric oxide.

## CHEMICAL COMPOSITION AND NUTRITIONAL CHARACTERISTICS

In spite of a long history of usage in the medicinal cuisine of oriental regions and countries, until approximately 20 years ago, only a limited number of studies have been conducted to verify the chemical composition and implicated functions of *N. commune*. These studies were mainly undertaken by Chinese researchers and reported in Chinese journals.

Following the early efforts on the putative health benefits of *N. commune*, recent studies have identified several components, including proteins, peptides (enzymes), amino acids, fatty acids, carbohydrates, vitamins, minerals, and other bioactive chemicals. Proteins, peptides, and free amino acids account for 25%~27% of the dry weight of *N. commune*, and even greater percentage in *Nostoc flagelliforme* and *Laver* [10, 14]. The amino acid composition, both protein-bound and free, is considerably diverse, similar to that of commonly consumed mushrooms. For example, it contains all types of essential amino acids for human health, and their proportion is appropriate to meet our needs, indicating that it has a favorable nutritional value [10, 15]. *N. commune* consists of 51.12% carbohydrates [10], including galactose, glucose, trehalose, fructose, xylose and polyhydric alcohol [14]. It should be noted that *N. commune* is dominated by extracellular polysaccharides (EPS) (~80% dry weight), and that its composition is being examined and verified [16, 17]. The fatty acids of *N. commune* are very low, accounting for only 0.21% of its dry matter [10, 18, 19]. *N. commune* also contains dietary fiber [5, 20]. In addition, *N. commune* can be characterised by great variety in both the number and type of constitutive minerals, and it is especially enriched with essential elements, such as iron (Fe), zinc (Zn) and calcium (Ca) (~4‰), at significantly higher levels than in *Lentinusedodes*, *Auriculariaauricula* and *Nostoc flagelliforme* [10, 14, 21, 22]. This constitution makes a big difference in the prominent health promotion of *N. commune* through synergistic and supplementary effect with other nutrient molecules [21]. Some vitamins have also been characterised and quantified. For example, the content of vitamin C is equal to that in the *Nostoc flagelliforme* [10, 19].

**Figure 3:**
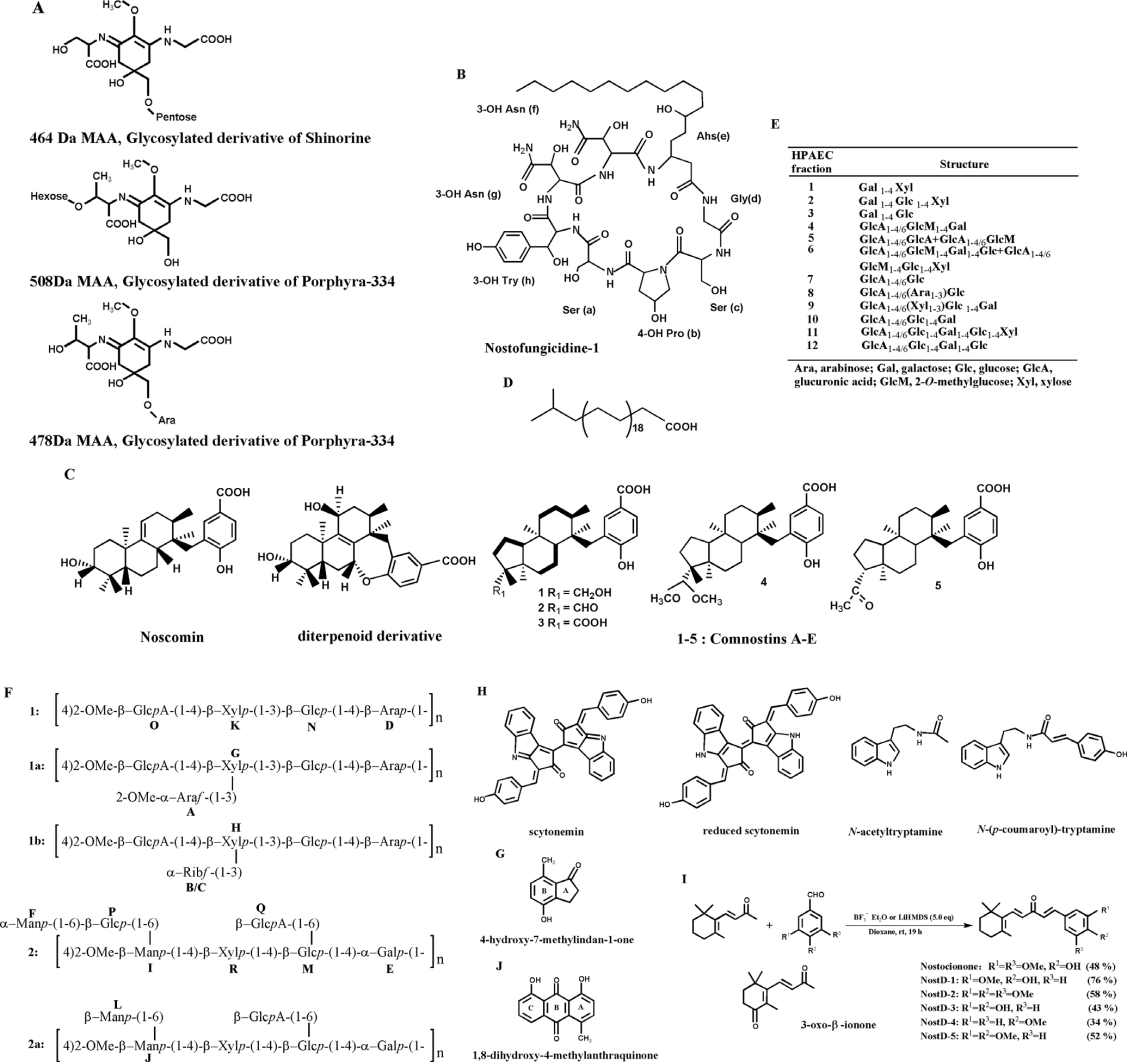
Chemical structures of common bioactive compounds from *N. commune* (**A**) Structure of glycosylated mycosporine-like amino acids [17, 49]. (**B**) Structure of an antifungal lipopeptide [33]. (**C**) Structure of diterpenoid derivatives which is uncommon in cyanobacteria [23–25]. (**D**) Structure of a long chain fatty acid [18]. (**E**) Structure of oligosaccharides isolated after weak acid hydrolysis of the acidic polysaccharide [11]. (**F**) Structure of Nc-5-s, a complex heteroglycan, composed of repeating units of 1, 1a, 1b and 2 and 2a in molar ratio of (10:25:50:5:10) [13, 66]. (**G**) Structure of an indane derivative [25]. (**H**) Structure of indole alkaloids [7, 8, 27, 41]. (**I**) Structure of β-ionone derivatives [4, 27, 41]. (**J**) Structure of an anthraquinone derivative [25].

Intriguingly, many phytochemicals and bioactive compounds purified from *N. commune* have been reported and identified, the structures of which are shown in Figure 3. These components could be divided into the following common types: (1) amino acids, fats and lipids (Figure 3A–3D), such as diterpenoid compounds, which are rather uncommon in cyanobacteria [23–25]; (2) carbohydrates (Figure 3E, 3F); (3) aromatic hydrocarbons and heterocyclic compounds (Figure 3G, 3H); (4) ketones and quinones (Figure 3I, 3J). Notably, most of compounds mentioned above have been only discovered and identified, and little is known regarding the details of potential functional properties. Taken together, *N. commune* is characterised by its great variety in both the number and type of nutrients, especially rich with various small molecules, comparable to those of *Auricularia auricular* [10, 17], making it a potential resource for natural functional foods.

It should also be noted that the distribution of these components varies as the cultural factors change during the growth, such as the origin, light, temperature, and nutritional levels. For example, it is unavoidable to mix with weeds and silt when *N. commune* is harvested in the fields [10]. The experimental methods, technology, determination of results and postharvest treatment could also lead to fluctuations and discrepancies [10, 17].

## ANTI-INFLAMMATORY AND ANTIBACTERIAL PROPERTIES OF *N. COMMUNE*

Inflammation is a natural immune response to injury or infection involving excessive production of inflammatory mediators, such as interleukin (IL) family cytokines, nitric oxide (NO), prostaglandin E2 (PGE2), ROS, and RNS, as well as the presence of highly activated inflammatory cells, such as neutrophils, monocytes and macrophages, that if unabated, lead to the development of serious chronic diseases, including cancer, cardiovascular disease, neurodegeneration and metabolic disorders [1, 26]. Extensive studies have shown that functional foods are appealing alternatives to clinical pharmacological therapies for treating and preventing inflammation and inflammation-related diseases.

Some important insights into the anti-inflammatory activity of *N. commune* were obtained based on cell culture studies. In a study conducted by Itoh *et al*, reduced scytonemin (R-scy) significantly suppressed LPS/IFNγ-induced NO production in murine macrophage Raw 264.7 cells. Mechanistically, R-scy was found to generate intracellular ROS, and thereby activate p38 MAP kinase and PI3K/Akt. The resultant Nrf2/ARE activation induces HO-1 expression and augments antioxidative activity, resulting in suppression of LPS/IFNγ-induced inflammatory responses [8]. They also found that nostocionone (Nost) and one of its derivatives (NostD3) significantly inhibit *Propionibacterium acnes* growth. Furthermore, they investigated the effects and detected that both Nost and NostD3 suppressed heat-killed *P. acnes*-induced nitric oxide (NO) production by suppressing of inducible NO synthase expression, following inactivation of NF-κ.B, and that NostD3 showed higher efficacy than Nost [27]. Hardardottir *et al*. reported that a heteroglycan, designated as Nc-5-s, had anti-inflammatory effects on IL-6 and IL-10 secretion by THP-1 monocytes primed with interferon-g and stimulated with LPS through the ERK1/2 pathway and/or the Akt/phosphoinositide 3-kinase pathway [13].

Recent studies have proved the antibacterial and antifungal properties of *N. commune* and *N. commune*-derived extracts. Bi *et al.* compared the antibacterial and antifungal properties of various extracts from *N. commune*, including petroleum ether, acetylacetic ester, acetone, ethanol, carbinol and aqueous extracts, and found that lower polarity ethyl acetate and acetone extracts exhibit potent antibacterial activity against *P. aeruginosa*, compared to those of *Eschenchia coil*, *Ahernaria alternate*, *Peniicilliumsp* and *Trichotheciumroseum*, whereas higher polarity methanol and ethanol extracts exhibit potent antibacterial activity against *Alternariaaltemata, Peniicilliumsp* and *Trichetheciumroseum*, compared to those of *Eschenchia coil*, *P. aeruginosa* and *Ahernaria alternate* [28]. Hassan and Li *et al.* found that the methanol extract from *N. commune* showed obvious inhibition of the growth of *Escherichia coli*, *Staphylococcus aureus*, *Bacillus subtilis*, *Moniliaalbican* and *Serratiamarcescens* (MIC: 1.5–1.0 mg/mL) [29, 30]. Yang *et al.* evaluated the antibacterial activity of the fat-soluble component and observed that it exerted better inhibitory effects against *Escherichia coli* (MIC: 64 μg/mL) [31]. In a continuing investigation, Sticher *et al.* reported several diterpenoid (Noscomin and Comnostins A-E), anthraquinone and indane derivatives, and found that Noscomin, Comnostin E and another diterpenoid had MIC values for *Staphylococcus epidermidis* equal to those of chloramphenicol, and the MIC of Comnostin C for *Escherichia coli* was equal to that of tetracycline. Other compounds only possess moderate antibacterial activity [23, 25]. Polysaccharides were shown to potently inhibit *Escherichia coli* and *Bacillus subtilis* (MIC: 50 μg/mL) by Li *et al* [32]. In addition, the antifungal activity of *N. commune* extracts was also demonstrated, including polysaccharides, total flavonoids and Nostofungicidine, which showed significant inhibition against *Aspergillus nigere*, *Candida albicans* and *Aspergillus candidus* [32–34].

The mechanism of the antibacterial action of *N. commune* and *N. commune*-derived extracts has remained unelucidated, although *N. commune* has been proved to be a powerful anti-bacterial and antifungal agent *in vitro*. Further research in animals and humans is therefore warranted to ascertain whether *N. commune* could attenuate inflammation or inflammation-associated diseases.

## ANTIOXIDATIVE PROPERTIES OF *N. COMMUNE*

Oxidative stress is provoked by a number of free radicals, including oxygen free radicals, ROS, and reactive nitrogen species (RNS), which are products of multiple cellular metabolic processes [35–37]. Normally, oxidative stress could be eliminated by endogenous antioxidases. However, once redox homeostasis is in disorder, radicals over-accumulate, resulting in DNA damage and inactivation of proteins and enzymes, which sequentially contribute to the onset and progression of mutagenesis, aging, inflammation, carcinogenesis and cardiovasculardiseases [36–39]. Considerable attention has therefore been devoted to the development and utilization of more effective non-toxic antioxidants of natural origin to improve human health.

The antioxidative capacity of the *N. commune*-derived extract was established *in vitro* based on its reducing capacity, the β-carotene survival rate and scavenging of 2,2’-azino-bis(3-ethylbenzothiazoline-6-sulfonic acid; ABTS) radical, superoxide anions (O_2_^·−^), Fe^2+^, peroxyl radicals, hydroxyl radicals, and peroxynitrite [40–50]. Compared to *Nostoc flagelliforme* and *Nostoc sphaeroide*s Kütz, the O_2_^·−^ scavenging capacity value of polysaccharides from *N. commune* was higher by 54.8% and by 103.7%, respectively [42]. Meanwhile, Huang *et al.* further detected that the polysaccharide can increase antioxidase activity, decrease the lipid peroxidation level, and reduce paraquat-induced oxidative damage using the model animal *Caenorhabditis elegans* [6]. In turn, it has been proved that the antioxidative ability of total flavonoids is higher than those of polysaccharides and fat-soluble components by Diao *et al.* [48, 50].

In addition, several glycosylated mycosporine-like amino acids (MAA) have been purified and identified from *N. commune*, such as porphyra-334 (346 Da), pentose-bound shinorine (464 Da), 7-O-(b-arabinopyranosyl)-porphyra-334 (478 Da), hexose-bound porphyra-334 (508 Da) and 1050-Da [17, 49]. When Sakamoto *et al*. directly monitored the decrease of ABTS radicals by ESR, the activity of the 478-Da MAA (IC_50_: 185 mM) was equivalent to that of Trolox (used for a standard with IC_50_: 182 mM), and the 1050-Da MAA (IC_50_: 55 mM) showed higher activity than did Trolox, contributing approximately 27% of the total radical scavenging activity in a water extract of *N. commune* [17]. MAAs are UV absorbing pigments and 478-Da MAA has an absorption maximum at 335 nm. In particular, the 1050-Da MAA with Amax at 312 and 340 nm, consists of two distinct chromophores of 3-aminocyclohexen-1-one and 1,3-diaminocyclohexen as well as two pentose and hexose sugars [17]. UVA (400–320 nm) radiation, which contributes to up to 95% of the total UV exposure, is not absorbed by DNA but it is a strong oxidant and considered the most important source of oxidative stress [51]. Therefore, we structurally speculate MAAs are likely to protect against UV radiation by scavenging ROS, including excited singlet oxygen and triplet state molecules [51].

The reports described above demonstrate that *N. commune* possess beneficial and effective antioxidative activity, and identify compounds that are potentially responsible for these effects. However, it should be mentioned that different or even contrary outcomes from a type of extract obtained in the above-mentioned studies could be due to different methods and reaction systems for testing [44, 45]. In addition, we should note that the orientation and location of *N. commune* may contribute to the discrepancy [42, 47].

## ANTI-CARCINOGENIC AND IMMUNOMODULATORY PROPERTIES OF *N. COMMUNE*

Cancer is still one of the deadliest diseases and a major threat to human health and life worldwide. Current projections imply that the incidence of cases will increase by 70% over the next twenty years, further highlighting the critical need to identify safe and effective approaches to managing cancer [52]. Tumour metastasis is the most destructive stage of cancer progression and the leading cause of cancer-related death. It is a complex and multistep event, wherein cancer cells leave the site of the primary tumour and disseminate to distant sites in the body [53, 54]. Taking into account the serious side effects and toxicity of conventional chemotherapeutic agents, a great deal of researches has focused on discovering anticancer alternatives from natural resources for the development of effective and safe therapeutics.

In the early stage of *N. commune* research, it was believed that *N. commune* had anti-cancer activity, which was widely publicized. Not until 2007, was its anti-cancer activity successfully proved and verified by precise scientific experiments conducted by Zhang *et al*. The crude extract from *N. commune* was shown to significantly inhibit the proliferation of A549 and SMMC-7721 cells with IC_50_ of 24.79 and 51.33 μg/mL, respectively [43]. Recently, studies have demonstrated that a small molecule designated reduced scytonemin (R-scy) isolated from *N. commune* may be able to induce autophagic cell death in human T-lymphoid line Jurkat cells. These studies further examined the mechanisms where cells treated with R-scy produced large amount of ROS, leading to mitochondrial dysfunction [7]. In continuation of their studies, a compound, named Nostocionone (Nost), was isolated from *N. commune*, and its derivatives (NostDs) were synthesised. Interestingly, NostD3 showed more potent inhibition of cell growth than Nost in Jurkat cells. Mechanistically, NostD3 was found to potentiate cell apoptosis through promotion of the intrinsic apoptotic signal, accompanied by the release of endonuclease G (Endo-G) from mitochondria [4]. Subsequently, Guo *et al*. reported that the growth of DLD1 human colon cancer cells was more significantly blocked by water stress proteins (WSPs), with an IC_50_ of 0.19 ± 0.02 μg/mL, through a caspase-dependent pathway than other cell lines, including HCT-116, HT-29, and SW480. Meanwhile, WSP1 has also been shown to possess interference in G_1_/S arrest. Additionally, they also observed similar results *in vivo* in which BALB/c mice were subcutaneously implanted with DLD1 cells. Moreover, treatment with recombinant WSP1 (rWSP1) clearly reduced the proliferation of SW480 cells. Besides, they suggested that WSP1 exhibits anti-metastasis activity by weakening cell invasion of DLD1 cells through upregulating the levels of E-cadherin and reducing N-cadherin, vimentin and integrin β1 [55–57]. Polysaccharides have been implicated as major active components of natural drugs and agents, and they always possess strong anti-cancer properties [58, 59]. The results obtained from our study are in agreement with these observations. In our research, polysaccharides from *N. commune* (NVPS) were first shown to exhibit suppression of proliferation of MCF-7 human breast cancer cells and DLD-1 colorectal cancer cells (IC_50_ = 67 and 110 μg/mL, respectively). Further analysis indicated that NVPS-incurred cell growth inhibition was strictly related to apoptosis through intrinsic, extrinsic and endoplasmic reticulum stress (ERS)-mediated apoptotic signalling pathways [60]. We extended our investigation to elucidate anti-metastatic activity of NVPS in human small cell lung cancer cells (NCI-H446 and NCI-H1688). The results showed that NVPS remarkably suppressed cell migration by blocking the epithelial-mesenchymal transition by abating integrin β1/FAK signalling through enhancing cell-matrix adhesion, increasing E-cadherin expression, and decreasing N-cadherin, vimentin, and MMP-9 expression, which resulted in the blockage of STAT3 nuclear translocation and JAK1 signalling [61].

Immunomodulatory activity has frequently been discussed as an indirect mechanism of anti-cancer potential [62, 63]. Generally speaking, α-glucan has little bioactivity, and most effective biologically polysaccharides main-chain structures contain β-(1→3)-*D*-glucan [64]. In 2000, Paulsen *et al* found that polysaccharides of *N. commune* from field material were shown to activate the complement system stronger compared with the standard polysaccharide, the acidic one the highest [11]. There is one major thing that all the polymers have in common: the higher amount of 1,3 substituted glucose units [11]. Liao *et al.* also reported the immunomodulatory potential of the polysaccharide-rich extract from *N. commune* (NCPS) and found that the immunity of murine macrophage Raw 264.7 cells might be activated by nitric oxide and superoxide secretion and consequently effectively inhibit the growth of human leukemic U937 cells as well as trigger the differentiation of U937 monoblast cells into monocytic/macrophagic lines resulting from the upregulation of GM-CSF and IL-1β as well as the downregulation of IL-6 and IL-17 [65]. Taken together, these studies demonstrated that *N. commune* may have potential as a cancer chemopreventative agent. However, the relationships between the structure and activity are still not fully understood yet.

## CONCLUSIONS

In addition to the broad spectrum of functional properties aforementioned, there are several additional effects of *N. commune* and *N. commune*-derived extracts have been reported, such as the hypocholesterolemic function [5, 20]. Therefore, the available reports may provide evidence that *N. commune* is distributed worldwide, as reviewed here, and is characterised by its great variety in both the number and type of nutrients, especially rich with various small molecules, making it an attractive natural resource treasure. Majority of current studies are concentrated on antioxidative, anti-inflammatory and antibacterial properties, rather other activities, providing broad research areas in future. Further research, especially human randomised clinical trials, is vital to ascertain the detailed beneficial effects, bioactivities and molecular mechanisms of *N. commune* and *N. commune*-derived extracts. We believe these efforts will call more attention to the value of *N. commune* on human health, and contribute to the development and application in functional foods and medicines targeted at the prevention and treatment of the mentioned above diseases.
